# Context-Aware Winter Sports Based on Multivariate Sequence Learning

**DOI:** 10.3390/s19153296

**Published:** 2019-07-26

**Authors:** Byung-Kil Han, Je-Kwang Ryu, Seung-Chan Kim

**Affiliations:** 1 Telerobotics and Control Laboratory, Korea Advanced Institute of Science Technology, Daejeon 34141, Korea; 2 Institute for Cognitive Science, Seoul National University, Seoul 08826, Korea; 3 Intelligent Robotics Laboratory, Hallym University, Chuncheon 24252, Korea

**Keywords:** deep learning, motion context, multivariate signals, recurrent neural network, sequence model, sequence model

## Abstract

In this paper, we present an intelligent system that is capable of estimating the status of a player engaging in winter activities based on the sequence analysis of multivariate time-series sensor signals. Among the winter activities, this paper mainly focuses on downhill winter sports such as alpine skiing and snowboarding. Assuming that the mechanical vibrations generated by physical interaction between the ground surface and ski/snowboard in motion can describe the ground conditions and playing contexts, we utilize inertial and vibration signals to categorize the motion context. For example, the proposed system estimates whether the player is sitting on a ski lift or standing on the escalator, or skiing on wet or snowy ground, etc. To measure the movement of a player during a game or on the move, we develop a custom embedded system comprising a motion sensor and piezo transducer. The captured multivariate sequence signals are then trained in a supervised fashion. We adopt artificial neural network approaches (e.g., 1D convolutional neural network, and gated recurrent neural networks, such as long short-term memory and gated recurrent units). The experimental results validate the feasibility of the proposed approach.

## 1. Introduction

Snow deposited on a slope is typically in a state of continuous deformation [1]. Ground conditions play a significant role in a variety of winter sports and recreational activities. For example, the preparation of equipment (e.g., the type and quantity of wax applied to the base of a set of skis or a snowboard) can vary depending on the snow quality (e.g., slush or ice). Also, surfaces with different snow conditions likely lead to skiers bumping or moving in unexpected ways, forcing them to adjust their riding styles on slopes. For example, when snow is melting (e.g., slushy snow in which the snow particles are completely immersed in water), snowboarders generally tend to move their center of mass backwards, such that the nose of their board does not collide with and stick to the wet ground. Although understanding snow quality is important, the identification of such physical characteristics through remote surveillance (e.g., computer vision techniques) is challenging or too costly to practically apply over large snowfields.

Besides estimating the quality of snow deposited on the ground, we focused on estimating a participant’s motion context, defined as the context that describes the participant’s situation or state in a snow field (e.g., skiing downhill and sitting on a ski lift). In fact, detecting and reporting conditions at ski resorts are important in that low temperatures may cause significant hurt to participants under abnormal snow conditions. In addition, quantification and classification of a player’s motion are important for coaching and prediction purposes from the perspective of sports analytics [2].

To estimate both ground conditions and motion contexts, we utilized inertial and audio signals, generated from friction and motion. These signals can describe dynamic information. To this end, we first collected time-series signals with a custom-built embedded system, which incorporates a number of sensors on board. Then, we trained the captured multivariate time-series signals in a supervised learning setting (i.e., a many-to-one mapping problem) using different types of machine learning techniques. This paper primarily contributes to the literature in two ways:

1. We proposed an intelligent context-aware system that classifies both ground conditions and motion contexts based on the incoming inertial and vibrational sensor measurements.
2. We conducted an experiment to investigate whether the proposed contexts can be classified based on the collected time-series signals.

The next section discusses related research.

## 2. Related Work

### 2.1. Sequence Classification in Human-Computer Interaction

Predicting a category of a given input sequence is called sequence classification, which forms the core of a variety of human–computer interaction applications [3,4,5,6,7]. Although a number of conventional approaches, such as k-nearest neighbor classifiers, Naïve Bayes, random forest [8], and support vector machine [9], are also widely used for sequence classification, the high feature dimensionality and sequential nature of signals whose feature attributes are ordered in time [10], complicate their understanding signals properly even when using sophisticated feature engineering techniques [11].

On the other hand, feedforward neural networks, which accept input data and learn complex features internally [12,13] for approximating some functions [14], are receiving growing attention for such tasks. Notably, recurrent neural networks (RNNs), which have recurrent hidden states that encode sequential information into a fixed length of vectors [15,16], have achieved state-of-the-art performance for a variety of sequence classification tasks [17,18,19]. Socher et al. proposed an intelligent system that classifies 3D objects in RGB-D (RGB and Depth) video sequences [17] by employing convolutional neural network (CNN) and RNN-based sequence learning architecture. RNN-based architecture was widely employed in the area of gesture recognition tasks to process RGB videos [20] and inertial sensor sequences from wearable devices [21,22]. Recently, Kim and Han revealed that a multidimensional temporal sequence can be encoded as latent space vectors using gated RNNs (e.g., long short-term memory (LSTM) [23] and gated recurrent units (GRUs) [15]) [24].

In a recent pioneering work [25], Yoon reported interesting results for a classification task of time-series signals (e.g., a natural language processing task) using CNN, which was originally introduced for a computer vision task approximately 30 years ago [26]. Because CNN is capable of learning both local and global features even from sequential data while allowing parallel computation [25,27] and requiring fewer pre-processing tasks, it is widely employed in many recent time-series applications [27,28,29,30,31].

### 2.2. Sensor-Based Sports Analytics

Internet of Things technologies are widely adopted for various sports analytics [2,32,33,34,35]. In a recent study, Yu et al. proposed an intelligent system that could analyze the inertial signals captured from sensors attached to multiple body parts of a professional skier [34]. In another recent study, Umek et al. designed a sensor-laden sports equipment for capturing the motion of a golf club [33]. They conducted an experiment with a set of sensors, such as a strain gauge, an accelerometer, and a gyroscope, to quantify the player’s motion. Wearable devices are also widely utilized for seamless sports analytics [32]. In a commercial market, a variety of sports activities, (e.g., basketball, tennis, soccer, running, etc.) are now being assisted by sensor and machine learning technologies [35]. For example, a motion and vibration sensor attached to the grip end of tennis racket can track a player’s shot type, such as serve, smash, and volley forehand [36].

## 3. Proposed System

This section describes the developed prototype hardware and context understanding system that classifies a player’s motion state (e.g., sitting on the ski lift or standing on the escalator, or skiing on the snow) and ground conditions based on the measurements. As described in Section 1, we assumed that the measured signals will vary depending on bumpiness of the surface.

### 3.1. Hardware

**Figure 1:**
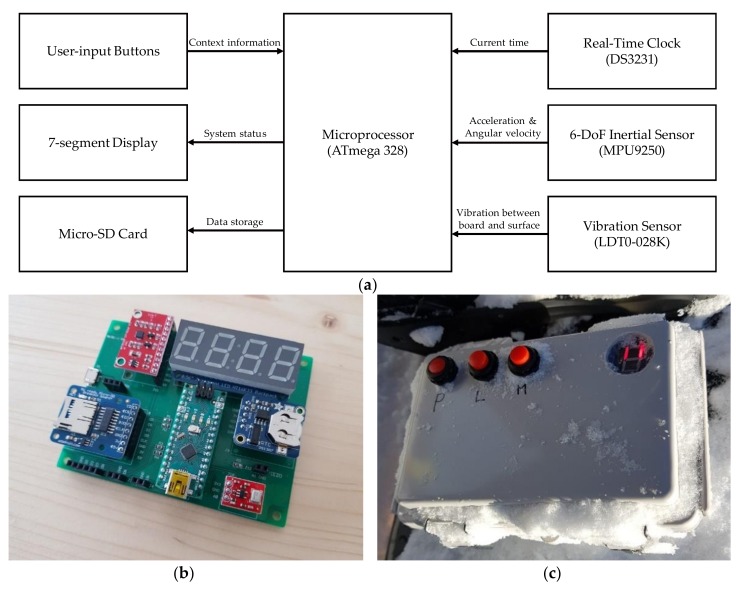
(**a**) Block diagram of the embedded system for data acquisition. (**b**) Developed prototype embedded system. (**c**) Developed prototype sensor box attached to the top of the snowboard deck. Three buttons (including one mode selection button) and a commercial seven-segment display were fitted to the device’s housing. A waterproof plastic case and push buttons were used. Photo (**c**) was taken after conducting a test run.

A multivariate time-series dataset was collected using a custom-built embedded system based on an ATmega328 microprocessor (Arduino Nano) with multiple sensors, namely a motion sensor (MPU9250, InvenSense, San Jose, CA, USA) and a vibration sensor (LDT0-028K, Measurement Specialties, Inc., Hampton, VA, USA), composed of piezoelectric polyvinylidene difluoride polymer film for capturing the ground vibrations generated mainly due to friction. To record the sensor signals and context information simultaneously, we also added a mode selection button on the outer cover of the sensor box (see Figure 1). A MicroSD card breakout board (Adafruit Industries, New York, NY, USA) and a real-time clock (RTC) with an integrated temperature compensated crystal oscillator (DS3231, Maxim Integrated, San Jose, CA, USA) were utilized for recording the data into the memory card. Then, we analyzed the recorded sequential data in a supervised manner. Figure 1 shows the developed prototype board.

### 3.2. Experiment-Sequence Classification

This section describes the process of the proposed sequence learning task. Using the sensor board described in the previous section, we collected data at 250 Hz at the Elysian Ski resort, Gangwon, South Korea, from a professional snowboard player, during February 2018. Table 1 shows the relevant weather information. The snowboarder who participated in this study tried to snowboard on the same route, preferably with the same strategy. We collected 2400 samples in total, each of which was comprised of 100 time steps. We balanced the data distribution by adjusting the number of data per class. The datasets were split such that 70% was used for training and the remainder for test purposes.

Considering that temperature and humidity determines the quality of the deposited snow on ground, we set five classes as described in Table 2.

**Figure 2:**
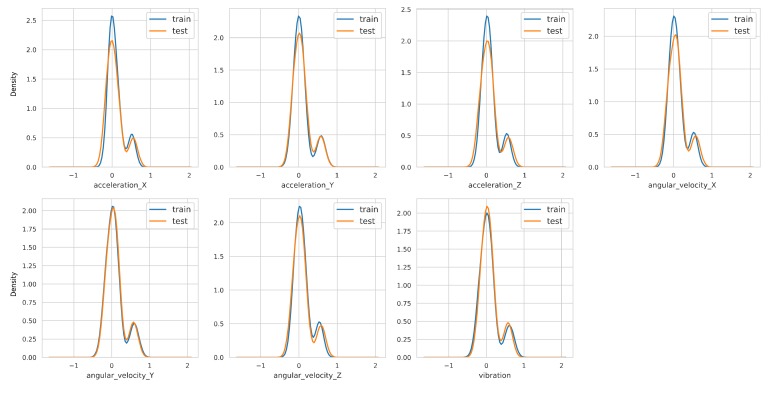
Visualization of the distributions of the training and test datasets based on kernel density estimation (KDE) to check if the split is stratified. The x-axis is the range of values in the dataset, and y-axis represents its probability density, which captures the underlying distribution of the sensor measurements. As shown, the distribution of the datasets for training and testing is similar.

To obtain a sense of how the measurements are distributed and to verify that the training and the test data are in the same distribution, we conducted an analysis using kernel density estimation (KDE). Figure 2 shows training and test dataset distributions.

**Figure 3:**
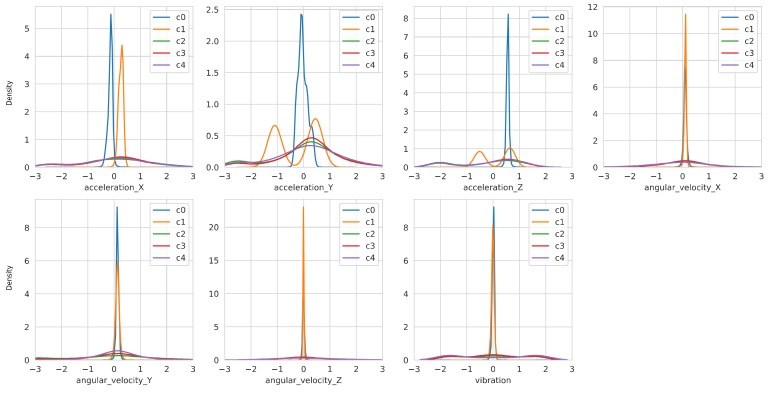
Density plots of variables in the training and test datasets with respect to classes. Differences in distributions among variables can be observed. The class index listed in the legend means the class index defined in Table 2.

In Figure 3, different colors represent the different values of classes defined in Table 2.

The density plots of variables in Figure 3 provides information regarding their distribution, such as the spread of the variables and central tendency. Especially, linear accelerations and angular velocities in Y-axis have apparently different probability density distributions.

#### 3.2.1. Baseline Classifier

As the baseline, we adopted a random forest classifier, which uses a large number of decision trees in the ensemble on various sub-samples of the dataset [8]. It often shows better performance (i.e., predictive accuracy) than a single decision tree in an aggregation of many decision trees, each of which may be prone to noise, because it often reduces the effect of noises [37,38]. Also, it generally demonstrates robust results against overfitting [39]. We adopted this classifier as a baseline because it does not consider the sequential nature of a given signal.

Based on the measured signals, we also generated three additional sequences, used for further feature engineering processes. Table 3 summarizes the name of the sequence used for this study and describes them.

Each sequence among the ten concurrent sequences (seven measured and three derived signals) was then used for calculating the following features: mean, median, min, max, max/min, std, skew, abs_max, min of abs, mean of abs, and standard deviation of abs, as summarized in Table 4. Therefore, 110 (11 sequences × 10 features) features were derived in total.

#### 3.2.2. One-Dimensional Convolutional Neural Network Model

**Figure 4:**
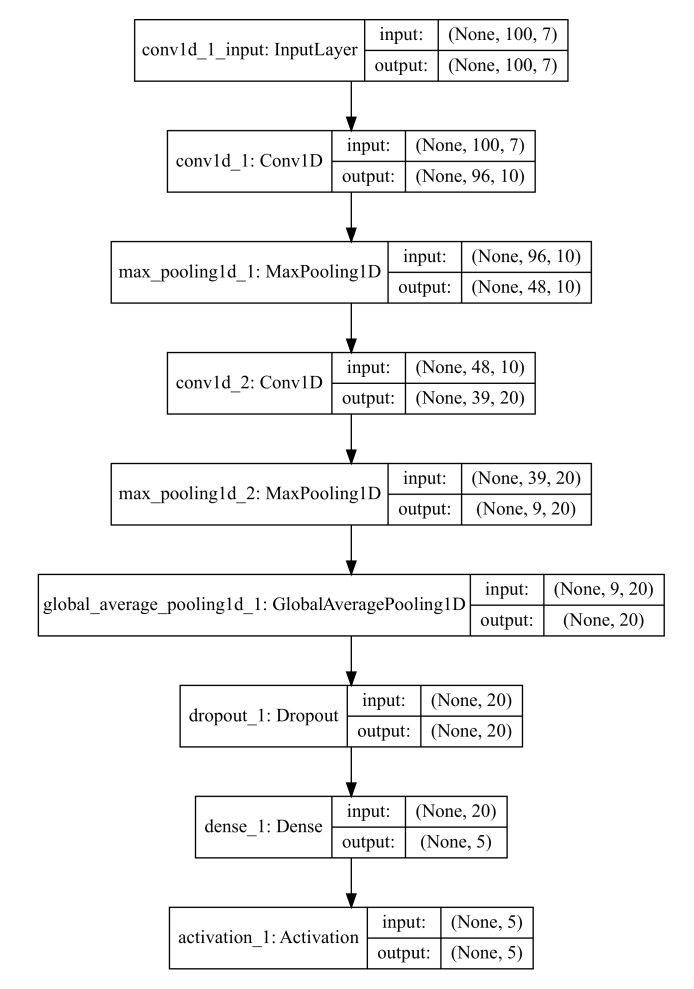
Example of the 1-D CNN model used for this study. Depending on the required performance and inference time, type/combination of input data and kernel size and numbers used at each layer may change.

We first adopted the 1D-CNN approach, in which a set of kernels is convolved with the inputs along a single temporal dimension as in [27]. We employed a sequential model implemented in Keras, which uses a linear stack of layers, for the training process. Figure 4 shows the model structure used for this study.

The convolution parts (e.g., Conv1D followed by MaxPooling1D) in the model depicted in Figure 4 learn how to extract features from the time-series sequences. Further, a fully connected layer (e.g., Dense) learns how to associate the learnt internal features to the types of motion context and overall surface quality listed in Table 2. As discussed in Section 2.1, the CNN-based approach has advantages in terms of parallel computation, in principle.

#### 3.2.3. Gated RNNs—LSTM and GRU

**Figure 5:**
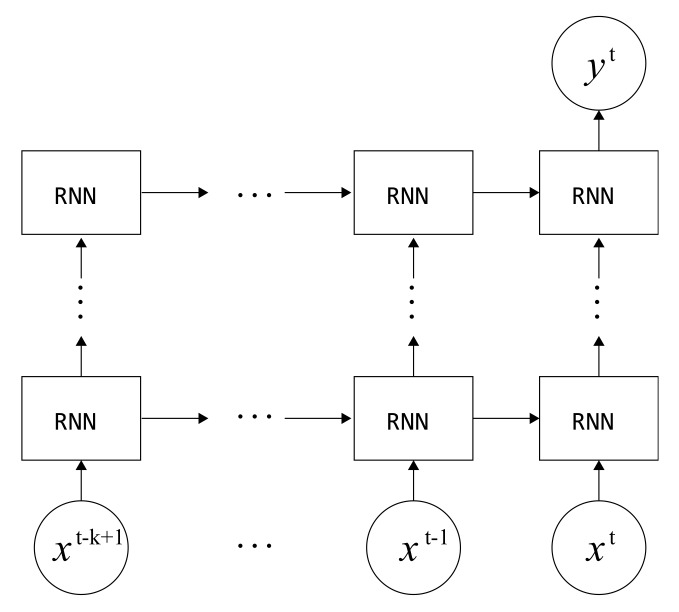
Recurrent neural networks (RNN)-based architecture used for the proposed motion context classification. An RNN cell is stacked on top of another one to learn more complex representations. In the experiment, long short-term memory (LSTM) and gated recurrent unit (GRU) cells were employed.

The basic RNN algorithm (i.e., RNN with a traditional tanh unit), especially one with a deep stacked architecture, poses the problem of vanishing and exploding gradients which complicates the learning of long-term dependencies. LSTM was proposed to resolve this problem as it can learn long term dependencies by utilizing the memory cell and gate units. Thus, LSTM has been adopted by many applications [40]. The memory cell stores information taken from the input and previous cells over a period of time. This information is controlled by the gate units, which are composed of the update gate, forget gate, and output gate. GRU has also been shown to outperform vanilla RNN in many applications, such as language modelling, torque generation [41], and others [24]. Compared to LSTM, GRU is composed of a smaller number of gates, and therefore, it often allows faster optimization progress than LSTM. Also, GRU performs better in smaller datasets [16]. Based on the previously described properties of gated RNNs, we constructed a classification network as seen in Figure 5.

We trained the captured data based on the network with the stochastic gradient descent algorithm using Tensorflow.

### 3.3. Experimental Results and Discussion

Table 5 shows the test accuracy obtained from the experiments. Here, RF (random forest) was used as the baseline classifier.

**Figure 6:**
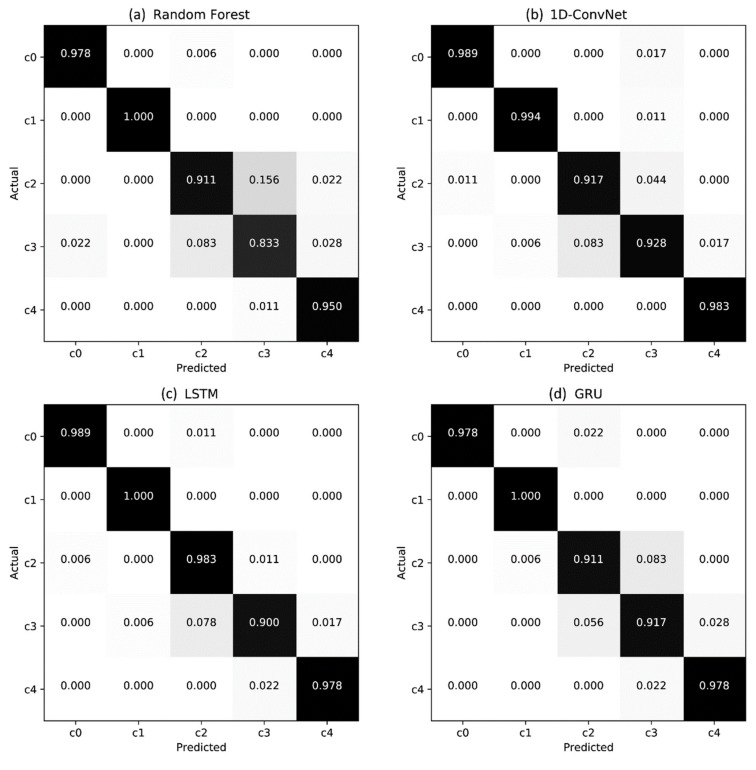
Confusion matrices of the classification results. Many misclassifications are between c2 (powder) and c3 (melt-freeze crust).

Overall, Table 5 shows that the sequence data from five different classes were reliably classified. Figure 6 shows a set of confusion matrices across all five classes when all measurements (i.e., dimension of 7) are used for inference.

Remarkably, the proposed model successfully identified not only motion contexts but also three different ground conditions affected by weather condition (e.g., powder, melt-free crust, and slush). The experimental results using fewer input signals (e.g., Acc + Gyro Y and Acc X + Gyro) also exhibited reasonable experimental results. This result can be used for reducing multiply-accumulates for high-speed/low-power inference operation during the runtime period.

## 4. Limitations and Future Work

### 4.1. Generalization of Our System

Thus far, we have proposed an intelligent sports system that understands contexts that are likely to occur during winter sports. We then validated the proposed system using various machine learning architectures. The results of the experiment validated the proposed approach.

However, owing to the nature of winter sports, experiments were not conducted under various conditions. Further, the trained model is overfitted to a single professional player; this is a disadvantage. Although our work validated the feasibility of the proposed approach, as future work, we plan to conduct additional experiments during the winter season to generalize our model.

### 4.2. Application to an Embedded System

**Figure 7:**
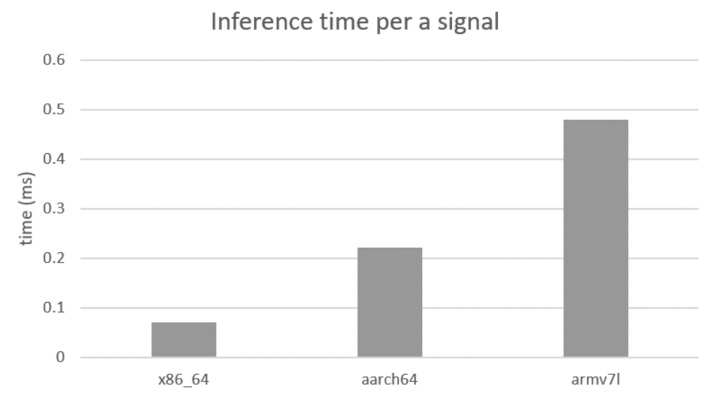
Inference time required for each signal (7 × 100 samples). From the left, the graph shows the time spent on PC (×86_64), NVIDIA Jetson TX2 (aarch64), and Raspberry Pi 3 Model B+ (armv7l).

The experimental results presented in this paper are derived from the signals stored using the Arduino-based embedded system, which later learned in the PC. In the future, we intend to implement this system in the embedded system for real-time inferences. To study its feasibility, we duplicated the trained model to other types of embedded machines such as Raspberry Pi 3 Model B+ (armv7l) and NVIDIA Jetson TX2 (aarch64) that supports an open source machine learning library (e.g., TensorFlow and Keras). Figure 7 shows the average inference time for a given sequence classification task with respect to the type of device.

Raspberry Pi 3 Model B+, which incorporates a 1.4 GHz 64-bit quad-core processor, costs approximately USD 50. Thus, the proposed approach can be implemented at the embedded device level.

### 4.3. User Interface for Labeling

Our prototype device assigns labels before motion takes place (i.e., by pressing a button outside of the box, as shown in Figure 1) due to the design characteristics of the system (i.e., it is supposed to be mounted on the board). Thus we faced difficulties in instant tagging, which is essential for capturing unexpected situations, such as a fall while skiing. As future work, we plan to develop a more user-friendly interface, capable of capturing contexts instantaneously.

## 5. Conclusions

In this paper, we proposed an intelligent system for winter sports that estimates status of a player engaging in winter activities based on the sequence analysis of multivariate time-series sensor signals. More specifically, the system classifies both ground conditions (e.g., powder, melt-free crust, and slush) and motion contexts (e.g., sitting on a ski lift or standing on the escalator) based on inertial and vibrational signals, assuming that surfaces under different snow conditions likely lead to skiers bumping into objects or skiing in different directions systematically.

We designed a set of sequence classifiers, such as random forest, convolutional neural network (1D-CNN), and gated recurrent neural networks (e.g., LSTM and GRU). The random forest classifier was utilized as the baseline. The experimental results validated the feasibility of the proposed approach. We expect that the proposed system will guide the design of smart systems for winter activities. In future studies, we plan to optimize the network structure to reduce the inference time so that the system can be used for real-time embedded applications.

## Figures and Tables

**Table 1:** Weather information of the ski resort.

	Date	Average Temperature	Relative Humidity	Daily Precipitation	Overall Quality
Day #1	7 February 2018	−10.6 °C (12.9 °F)	25.6%	-	Powder
Day #2	23 February 2018	1.9 °C (32.4 °F)	81.1%	5.2 mm	Melt-freeze crust
Day #3	26 February 2018	3.1 °C (37.6 °F)	43.1%	-	Slush

**Table 2:** Class used in the experiment.

Class Index	Motion Context (and Overall Surface Quality)
Class #0	Sitting on the lift
Class #1	Standing on the escalator
Class #2	Downhill on Day #1 (powder)
Class #3	Downhill on Day #2 (melt-freeze crust)
Class #4	Downhill on Day #3 (slush)

**Table 3:** Signals used in this study.

	Sequence Name	Descriptions
measured	acceleration_X	linear acceleration in X axis in m/s^2^
	acceleration_Y	linear acceleration in Y axis in m/s^2^
	acceleration_Z	linear acceleration in Z axis in m/s^2^
	angular_velocity_X	angular velocity in X axis in rad/s
	angular_velocity_Y	angular velocity in Y axis in rad/s
	angular_velocity_Z	angular velocity in Z axis in rad/s
	vibration	vibration intensity values
generated	total_linear_acceleration	root mean square of the linear acceleration in m/s^2^
	total_angular_velocity	root mean square of the angular velocity in rad/s
	acc_vs_vel	ratio of total_linear_acceleration and total_angular_velocity in m/(rad·s)

**Table 4:** Features explored in this study.

Feature Name	Descriptions
mean	arithmetic mean (average)
median	median
min (max)	minimum (maximum)
max/min	ratio of max and min
std	standard deviation
skew	sample skewness
abs_min	minimum of absolute value
abs_max	maximum of absolute value
abs_mean	arithmetic mean of absolute value
abs_std	standard deviation of absolute value

**Table 5:** Experimental results—Test accuracy (%).

Input Signals	Dim.	RF	1D-CNN	GRU	LSTM
Acc XYZ	3	77.25	77.03	80.60	77.69
Gyro XYZ	3	83.01	92.41	92.38	83.66
Acc + Gyro	6	91.91	96.11	96.24	95.63
Acc + Vib	4	87.25	87.67	90.33	85.55
Acc + Gyro Y	4	91.13	95.22	95.44	94.78
Acc X + Gyro	4	91.12	94.44	96.33	94.11
Acc + Gyro Y + Vib	5	93.53	94.89	95.00	94.22
All	7	93.44	96.22	95.67	97.00
